# Recombinant IL-21 induces perforin and granzyme B in total and virus specific CD8 Tcells in acute and early stages of SIV infection in rhesus macaques

**DOI:** 10.1186/1742-4690-9-S2-P13

**Published:** 2012-09-13

**Authors:** S Pallikkuth, L Micci, Z Ende, K Rogers, G Silvestry, F Villinger, M Paiardini, S Pahwa

**Affiliations:** 1 University of Miami, Miami, FL, USA; 2 Yerkes National Primate Research Center, Emory University, Atlanta, GA, USA; 3 University of Miami Miller School of Medicine, Miami, FL, USA

## Background

We have recently demonstrated that the cytokine IL-21 enhances the cytotoxic potential of CD8 T cells in chronically SIV infected rhesus macaques (vaccines 2011).

## Methods

In this study, 12 RM were infected with SIVmac239 (i.v., 300 TCID50). rMamu IL 21-Fc fusion protein (50mg/kg) was given s.c on a weekly basis post infection (pi) for 5 doses on days14, 21, 28, 35 and 42pi to 6 animals, designated as “treated”, with 3 mamuA01+ animals each in treated and control groups. Samples of PBMC, bone marrow (BM), rectal biopsy (RB) and peripheral LN (LN) were collected before infection (d-11), and at various times post infection.

## Results

Compared to controls, IL-21 treated animals demonstrated increases in frequency and MFI of Perforin (Perf) and granzyme B (GrB) at d45 in total CD8 T cells in PBMC, LN and RB, particularly in CM and Effector subsets; these were sustained up to d70pi. Perf and GrB levels increased in virus specific Tet+ CD8 T cells at d 45 in PBMC (p=0.029), LN (p=0.015), and RB (p=0.024). In the CD4 T cells, GrB induction was more prominent in the PBMC, LN, BM and RB. Frequencies of CD4 (p=0.011) and CD8 (p=0.031) CM T cells increased in PBMC at d70pi. T cell inhibitory molecule PD-1 and proliferation marker Ki67 were similar in treated and control animals. In treated animals, 2/6 showed a decline in post-peak viremia that was sustained up to d70pi follow up.

## Conclusion

In summary, IL-21 given s/c to SIV infected RM during early stages of infection led to augmented T cell cytotoxic granules perf and GrB in total and virus specific CD8 T cells in various anatomical sites. IL-21 should be explored further in vaccine strategies as an immunomodulating adjuvant.

